# Anterior and Posterior Tongue Regions and Taste Papillae: Distinct Roles and Regulatory Mechanisms with an Emphasis on Hedgehog Signaling and Antagonism

**DOI:** 10.3390/ijms24054833

**Published:** 2023-03-02

**Authors:** Archana Kumari, Charlotte M. Mistretta

**Affiliations:** 1 Cell Biology and Neuroscience, Rowan University School of Osteopathic Medicine, Stratford, NJ 08084, USA; 2 Department of Biologic and Materials Sciences & Prosthodontics, School of Dentistry, University of Michigan, Ann Arbor, MI 48109, USA

**Keywords:** fungiform papilla, circumvallate papilla, taste, taste bud, taste bud progenitors, Hedgehog signaling, Hedgehog antagonism, sonidegib, chorda tympani nerve, glossopharyngeal nerve

## Abstract

Sensory receptors across the entire tongue are engaged during eating. However, the tongue has distinctive regions with taste (fungiform and circumvallate) and non-taste (filiform) organs that are composed of specialized epithelia, connective tissues, and innervation. The tissue regions and papillae are adapted in form and function for taste and somatosensation associated with eating. It follows that homeostasis and regeneration of distinctive papillae and taste buds with particular functional roles require tailored molecular pathways. Nonetheless, in the chemosensory field, generalizations are often made between mechanisms that regulate anterior tongue fungiform and posterior circumvallate taste papillae, without a clear distinction that highlights the singular taste cell types and receptors in the papillae. We compare and contrast signaling regulation in the tongue and emphasize the Hedgehog pathway and antagonists as prime examples of signaling differences in anterior and posterior taste and non-taste papillae. Only with more attention to the roles and regulatory signals for different taste cells in distinct tongue regions can optimal treatments for taste dysfunctions be designed. In summary, if tissues are studied from one tongue region only, with associated specialized gustatory and non-gustatory organs, an incomplete and potentially misleading picture will emerge of how lingual sensory systems are involved in eating and altered in disease.

## 1. Introduction

From embryonic derivation into adulthood, the tongue has distinct regions, and these are exemplified by specialized innervation, vasculature, stroma, muscles, glands, and epithelium, with organs that are gustatory and non-gustatory [1,2]. The regions are adapted by form, tissue, and molecular signature to perform oral–pharyngeal functions that include eating, speaking, host protection, maintenance, and repair. These major functions in turn require the following: taste, touch, temperature, and pain sensations; mastication; swallowing; tissue turnover and recovery; and salivary gland secretion. Therefore, regulation of development, homeostasis, and regeneration in the specific lingual regions must require tailored molecular pathways.

In this paper, our focus is on sensory functions of the tongue associated with eating, with attention to lingual gustatory organs. The mammalian tongue, at the gateway to the oral cavity, has the ability to trigger acceptance of nutrient-rich food and rejection of spoiled food and poisons [3]. The sensory taste receptor cells that detect and recognize chemicals to determine ingestion or rejection are localized within three specialized taste organs, fungiform papillae (FGP) on the anterior two-thirds of the tongue, circumvallate papillae (CVP) on the posterior tongue at the oral–pharyngeal tongue border, and foliate papillae (FOP) on the posterolateral tongue on each side. Anterior FGP are derived from ectoderm, whereas posterior CVP and FOP are derivatives of endoderm [4]. Here, we will emphasize study of the FGP and CVP. Further, the principal attention is on mouse and rat tongues. Although incorporating human studies is out of scope here, we note that there is an emphasis on human tongue physiology in the recently published literature [5].

**Figure 1:**
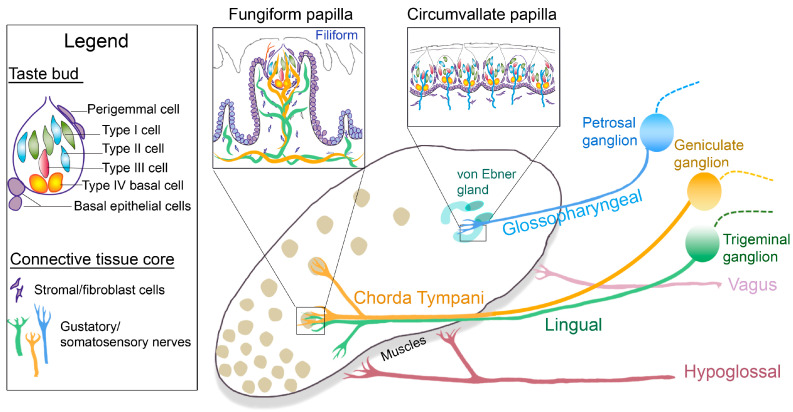
Anterior and posterior tongue papillae and nerves: Diagram of the tongue dorsum illustrating the taste organs, multiple fungiform (rounded) and single circumvallate (U-shaped) papillae on the anterior and posterior tongue, respectively. Minor salivary von Ebner glands, associated with circumvallate papillae, are labelled in the posterior tongue. The gustatory nerves, chorda tympani (yellow) and glossopharyngeal (blue), project from the corresponding sensory ganglia, geniculate and petrosal, respectively, to the anterior and posterior papilla taste buds. The somatosensory lingual nerve (green) fibers derive from the trigeminal ganglion, enter the anterior tongue together with the chorda tympani, and are within the connective tissue core of the Fungiform and non-gustatory Filiform papillae. The central projections of these nerves from the ganglia are represented in dashed lines. Hypoglossal and vagus motor fibers innervate the extrinsic and intrinsic tongue muscles and the posterior palatoglossus muscles. The boxed diagrams are as follows: The fungiform papilla, surrounded by non-taste filiform papillae, includes a single apical taste bud and a broad connective tissue core with stromal cells and innervation. The circumvallate papilla has numerous taste buds aligned next to each other in the epithelium with innervation and stromal cells in the connective tissues. The legend includes taste bud cell types (Type I, II, and III), taste bud progenitors (Type IV basal cells, perigemmal cells, and basal epithelial cells), and elements of the connective tissue core.

FGP are scattered on the anterior tongue in a patterned array, highly dense on the tip, and are surrounded by the non-taste filiform papillae (Figure 1). In mice and rats, the FGP houses a single apical taste bud (TB) in the epithelium and enfolds a broad connective tissue core with stromal cells, matrix, and innervation (Figure 1, inset Fungiform papilla). The CVP, however, is single in number but has multiple and contiguous TB in the papilla epithelial walls (Figure 1, inset Circumvallate papilla). Further, the CVP epithelium surrounds a connective tissue core and is continuous with von Ebner glands at the papilla base. Von Ebner glands, a subset of the minor salivary glands, secrete a serous fluid that is essential for taste perception [6], and they are unique to the CVP and posterior tongue. Thus, not only location and origin, but also the structure of FGP and CVP are distinctive.

The tongue, including FGP and CVP, is extensively innervated by distinct nerve fibers to transduce chemosensation and somatosensation. The gustatory nerve fibers transmit taste sensations to the brain from tongue taste cells that receive sensory information from external stimuli. The tongue TBs within FGP and CVP are innervated by gustatory chorda tympani (CT) or glossopharyngeal (GL) nerve fibers (Figure 1). In addition, the anterior tongue and FGP are innervated by lingual (LN) nerve fibers (Figure 1), which are predominantly somatosensory in nature. The CT nerve also extends fibers outside of the TBs, into the apical epithelium for receiving somatosensory information [7,8]. 

The CT and LN nerve afferents arise from the geniculate and trigeminal ganglion neurons, respectively, and enter the tongue in a common bundle but redistribute to taste (CT) and non-taste (LN) tissues (Figure 1). On the other hand, the posterior tongue GL nerve projections, which also convey somatosensory information [9], arise from petrosal ganglion neurons (Figure 1). Thus, both CT and GL nerve fibers are multimodal and can transduce taste, touch, and temperature sensations [8,10]. Recent studies have focused on the geniculate ganglion and CT nerve to identify somatosensory neurons [11,12,13,14,15], but parallel information from the petrosal ganglion and GL nerve has yet to be investigated. 

In addition, the tongue receives both sympathetic and parasympathetic nerves of the autonomic nervous system to perform salivary functions [16]. The LN nerve receives parasympathetic fibers from the CT [17], and the GL nerve includes sensory, motor, and parasympathetic fibers [18,19]. Tyrosine hydroxylase, a marker of peripheral sympathetic neurons, is also shown in CVP and FGP [20,21]. Further, the tongue has a distinct musculature that is supported by motor innervation. The extrinsic and intrinsic tongue muscles are innervated by the hypoglossal nerve, and the palatoglossus muscle at the posterior tongue is innervated by the vagus nerve [9,22] (Figure 1). Overall, in addition to gustatory and somatosensory nerve fibers in the anterior and posterior regions, the tongue has distinct sympathetic, parasympathetic, and motor innervations to perform taste, somatosensory, and motor functions. 

Interestingly, although the lingual regions are distinctly different, studies of peripheral lingual gustatory system function and regulation have often generalized between TBs in the posterior tongue and those in the anterior. However, our previous work established a differential Hedgehog (HH) signaling regulation of FGP and CVP in mouse taste organ homeostasis and regeneration [10,23]. Further, taste responsiveness to sweet, salt, sour, bitter, and umami stimuli varies between CT and GL taste nerves [10,24]. The difference in TB location, cell types, numbers, and molecular signatures along with different innervation and ganglia presumably contribute to variability in the transmitted sensory information. In mouse and rat models, because TBs are in larger numbers and have a focused location in CVP, these buds are often studied for cell and molecular studies, including gene expression, compared to the FGP, which are scattered and smaller in size and have a single TB. In contrast, for functional analyses, the CT nerve is a rational choice for electrophysiological recordings because of the comparatively better access for surgery than the GL nerve. However, individual anterior or posterior tongue tissue and nerve recordings cannot completely represent the respective overall picture of the tongue TBs and sensory responses. 

There have been several focused reviews covering aspects of taste development and transduction, communication between TBs and nerves, and TB regeneration during homeostasis as well as its disruption by cancer treatments [1,2,25,26,27,28,29]. Thus, for this review, our aim is to bring to light the cellular and molecular differences or similarities between the two lingual taste organs to better understand the function of distinct taste organs as well as to present ideas that include both organs for interpreting a whole mouth sensory effect. 

## 2. Taste Buds

The taste cells are tightly grouped in a garlic-shaped bud in FGP and CVP, with innervation from the geniculate and petrosal ganglia, respectively, to relay chemosensory information to the brainstem. In mice, TB size in terms of length is similar between FGP and CVP, but the width within FGP was observed to be larger than within CVP in mouse [30] and rat [31] models. This might be due to the different numbers of TBs that are housed within the two taste organs or the different numbers of taste cells that are within a TB. Recently, however, using whole mount tongue staining with a taste cell marker, K8, no differences in TB volume were observed between FGP and CVP [32], suggesting similar TB sizes. 

The TB is a heterogeneous collection of taste cells. Based on the membrane receptors and channels, taste cells are historically categorized into three types (Figure 1, Table 1). In addition to morphological differences, the three TB cell types are functionally different and are differentially sensitive in detecting specific taste stimuli [33]. Relative specificity in responding to a particular taste quality contrasts with the studies that emphasize that a TB cell is responsive to stimuli representing one or more of the taste qualities and with varying strength [34,35,36,37]. Further, there are differences in CT and GL nerve responses to the same tastant [10]. There would presumably be differences in the anterior FGP and posterior CVP taste cell molecular composition leading to the ability to respond to more than one stimulus with varying strength. 

Some of the major molecular components of taste cell types are summarized in Table 1, although we have not attempted comprehensive citations from all of the related literature. Briefly, the three cell types are: Type I taste cells. They are the most numerous cell type of the TB, including about half of all taste cells, and are glial-like support cells that wrap around other taste cells [38]. Because of the wrapping appearance, and thus the difficulty to discriminate from other cell types in whole mounts, limited data are available on their molecular composition/transcripts. The data from Table 1 suggest that Type I taste cells in FGP and CVP have only some overlap in molecular composition and in their sensitivity to amiloride. Type II taste cells. About one-third of the TB cells are Type II cells, and they are larger in diameter than Type I cells [33] irrespective of their location. They are elongated cells with multiple microvilli at the apex and membranous voltage-gated calcium channels and G-protein-coupled receptors (GPCR). Tastant-induced stimulation of GPCRs activates G-proteins and downstream PLCβ2, IP3R3, and TRPM5 pathways. In addition, the TRPM4 channel is identified in CVP and was shown to work together with TRPM5 [39]. Type II cells transduce sweet, bitter, and umami taste [40]. However, their taste responsivities are not uniform. For example, Tas2rs contribute to the detection of bitter taste and noxious chemicals [41] and are predominant in CVP [42], which might support the CVP in serving as a final checkpoint before any spoiled food enters the esophagus. Type III taste cells. Type III taste cells are the least frequent and have a single apical microvillus. They have proton-selective channels and express several synaptic proteins and thus are demonstrated as presynaptic cells. Anterior FGP Type III cells respond more specifically to sour stimuli [43] than CVP cells, which can respond to multiple taste stimuli [44,45,46]. Recently, the presence of a new subtype of Type III taste cells in both FGP and CVP has been suggested, which can broadly detect sour, bitter, sweet, and umami stimuli [47]. Type III cells are more numerous per TB in posterior CVP and more immunohistochemically diverse compared to those in anterior FGP [30,43,48,49]. The data in Table 1 indicate that there are increased proportions of Type III taste cell markers in CVP and that there may be more than two subtypes of Type III cells within the CVP. Overall, the overlapping or specialized molecular signatures of a taste cell type might associate with the differences in taste responsiveness observed between CT and GL nerves in FGP and CVP. 

**Table 1:** Molecular components of taste bud cell types in fungiform and circumvallate papillae.

Taste Cell Type	Taste Detection	Channel/Receptor/ Molecular Marker	Fungiform Papilla	Circumvallate Papilla
Type I	Salt	Epithelial sodium ion channels	65% Amiloride-sensitive [50]	Amiloride-insensitive [50,51]
			Comprised of α, β, and γ subunits [51]	50% cells do not have α, β, and γ subunits [51]
			Rich in β and γ subunits [51,52]	α subunit is predominant [52]
Type II	Sweet (Tas1r2 + Tas1r3) and umami (Tas1r1 + Tas1r3)	GPCR Tas1r1	Predominant expression [53,54,55]	Rare expression [53,54,55]
		GPCR Tas1r2	Rare expression [53,54]	Predominant expression [53,54]
		GPCR Tas1r3	In all of α-gustducin-expressing cells [53,56]	In 10% of α-gustducin-expressing cells [56,57]
	Bitter and toxic	GPCR Tas2rs	Rare expression [42,58]	Predominant expression [42,58,59]
	Sweet, bitter, umami	G protein subunit, Gustducin	α subunit is predominant [31]	α14 subunit is predominant [60,61]
		Galectin-3	In 66% of α-gustducin-expressing cells [42]	In 6% of α-gustducin-expressing cells [42]
		Receptor IP3R3 and PLCβ2	Expressed [62,63]	Expressed in increased proportions [30]
		Transient receptor potential (TRP) channel subfamily TRPM5	A few TRPM5+ cells do not have the Type II cell electrophysiological properties [48]	Always have the Type II cell electrophysiological properties [48]
Type III	Sour	TRP channel member PKD2L1	Expressed, its deletion reduced response to sour stimuli [64,65]	Expressed in increased proportion, deletion has no effect on sour response [64,65]
		TRP channel member PKD1L3	Expression not observed [64,65]	Expressed [64,65]
		Synaptic protein SNAP25	Expressed, a subset lacks PKD2L1 [49]	Expressed in increased proportion, highly coincident with PKD2L1 [49]
		GAD67 (for neurotransmitter GABA synthesis)	Expressed, significant subsets without PKD2L1 or SNAP25 [43,49]	Expressed in increased proportion, coincident with PKD2L1 or SNAP25 [49]
		Synaptic protein Car4	Co-expressed with SNAP25 and GAD67 [66]	A subset lacks SNAP25 and GAD67 [66]
		Neural cell adhesion molecule (NCAM)	Car4-NCAM+ in Type III cells, also co-labels with Type II marker IP3R3 [67]	Expressed only in Type III cells [67]

Furthermore, in addition to the above mentioned three taste cell types, there is another cell type at the base of the TB. Type IV basal cells within TBs are considered to be an intermediate form between the TB progenitor perigemmal cells and taste cells [68]. They are post-mitotic, similar to taste cells, and essential for TB differentiation as a progenitor cell [69,70,71]. The Type IV basal cells are discussed below in the section on **TB progenitors**. 

## 3. TB Progenitors 

**Figure 2:**
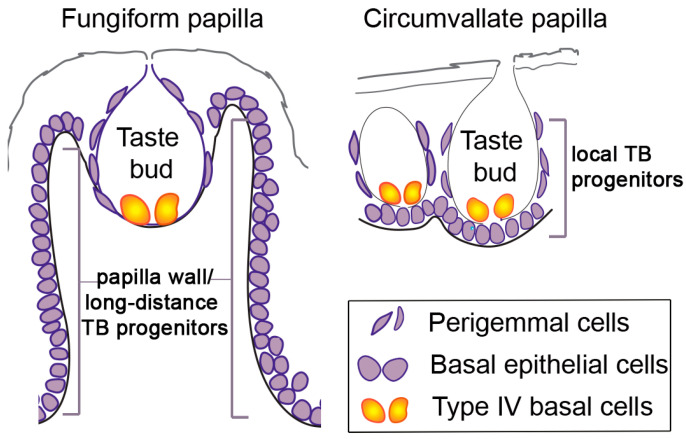
Taste bud (TB) progenitors. Different TB progenitor populations are demonstrated in the fungiform and circumvallate papillae: Type IV basal cells are within the TB; perigemmal cells are positioned outside of the TB; basal epithelial cells line the entire basement membrane of the papillae and are more locally positioned in the circumvallate papilla as compared to long-distance cells in the fungiform papilla wall.

Despite various differences in TB cell types, all taste cells in FGP and CVP are continuously renewed throughout life [69,72]. Taste cell life spans are distinct and range from about 8 to 40 days in CVP [71,72,73]. Parallel studies for FGP taste cell lifecycle are lacking. For continuous replacement of the dynamic taste cells, actively proliferating and tightly regulated TB progenitors are essential. Perigemmal cells, located outside TB and basal epithelial cells in both FGP and CVP (Figure 1 and Figure 2), serve as TB progenitors [68,69,74,75]. Local perigemmal and epithelial stem cells enter the TB as immature taste cells and differentiate as they move inside [71,76]. These immature taste cells are Type IV basal cells within TBs (Figure 2), which have lost the capacity to divide and become immediate taste cell progenitors. It takes about 12–18 h for an immature taste cell to enter into the TB after birth in both FGP and CVP [77] and about 2.5–3 days for a Type IV basal cell to differentiate into a mature TB cell [71,73]. In addition to these local TB progenitors, taste cells in FGP are also progeny of stem cells located within the papilla walls (Figure 2) [74,75]. These anterior tongue additional TB progenitors are at longer distances than the corresponding CVP basal epithelial TB progenitors (Figure 2) and thus might take a longer time to enter into the TB. Furthermore, TB progenitors in both FGP and CVP can give rise to adjacent non-taste filiform cells or keratinized epithelial cells, respectively [74,75]. Importantly, whereas the basal epithelial wall in FGP holds a number of TB progenitor cells, there is only a single TB per FGP in mice and rats, in contrast to the multiple TBs in the CVP (Figure 2).

In the posterior tongue, Leucine-rich repeat-containing G-protein-coupled receptor (LGR) 5+ and LGR6+ cells mark TB progenitors in CVP [78,79,80]. A parallel role in FGP is not observed. Further, LGR5 is not present in the adult FGP [79]. LGR5 and -6 are members of the GPCR family [81,82] and potentiate the canonical Wnt signaling pathway. However, it remains unclear whether LGR5 and LGR6 are co-expressed in CVP. Further, *Lgr5*+ cells seem to be a subset of *Sox2*+ basal epithelial TB progenitor cells in CVP [83]. The transcription factor, SOX2, regulates both anterior FGP and posterior CVP basal epithelial cells for TB differentiation [75,83,84]. In addition, the progeny of *Sox2*+ cells are also observed in the non-taste filiform epithelium next to FGP or keratinized epithelial cells in CVP [75,85]. However, *Sox2* is dispensable for maintaining homeostasis of non-gustatory papillary epithelium in CVP [83] but is indispensable for non-taste cell differentiation in the anterior tongue [84]. Recently, utilizing *Sox2* multi-colored lineage tracing, multiple stem cells were shown to contribute to a single TB in both FGP and CVP [86]. The authors further suggested that TB progenitor cells are maintained by turnover and might not be a long-lived stem cell. SOX2 is further regulated by Hedgehog (HH) signaling in taste papilla functions [84].

An extensive body of work has identified the essential roles of HH signaling and Sonic HH (SHH) ligand in taste cell production, maintenance, and renewal (reviewed in [1,2,87]). HH-responding *Gli1*+ cells are active in the TB progenitor population in both FGP [74] and CVP [88]. However, a transcription factor and HH pathway target, *Nkx2.2,* was observed only in CVP, and *Nkx2*.2-expressing cells gave rise to Type III taste cells [89]. Another transcription factor and a HH pathway target*, Ascl1* (*Mash1*), is present in both rat FGP and CVP epithelium [90] and co-expressed with *Nkx2.2* in CVP [91]. *Mash1* is well-studied in mouse CVP only and shown to differentiate predominantly into Type III cells and to a lesser extent to Type II cells [92,93,94,95]. Interestingly, *Mash1*-expressing cells present in the surrounding epithelial cells outside of TBs do not function as TB progenitors [95]. GLI3, a HH pathway repressor, was found in both FGP and CVP TB, co-expressed with Type II taste cells [96]. In CVP, *Gli3* functions as a repressor of the Tas1r3-expressing subtype of Type II taste cells [96]. In addition to the HH pathway, there are other signaling pathway transcription factors that are involved in the generation and differentiation of taste cell types in FGP and/or CVP and a few are noted here.

The Wnt signaling transcription factor, β-catenin, can drive differentiation of all cell types depending on its levels in TB progenitor cells [97,98]. β-catenin induces differentiation of predominantly Type I and II taste cells in CVP but only Type I in FGP [98]. In contrast, its stabilization in *Shh*+ Type IV basal cells promotes TB autonomous Type I cell differentiation in both FGP and CVP and additionally via signals extrinsic to TBs in CVP [98]. Further, inhibition of β-catenin caused greater loss of TBs in FGP than CVP [99]. Although SHH is shown to be a negative regulator of β-catenin signaling [100], loss of β-catenin was associated with reduction or elimination of SHH in FGP or in CVP, respectively [99]. Overall, the Wnt/β-catenin pathway differentially regulates FGP and CVP TBs. Separately, perigemmal TB progenitors within FGP and CVP expressed slowly dividing BMP4+ cells, which are additionally expressed in differentiating taste cells in CVP, further suggesting divergent pathways for taste cell differentiation in CVP as compared to FGP [77].

The transcription factor *Skn-1a/Pou2f3* plays an important role in the differentiation, specification, and maintenance of CVP Type II cells [101,102]. Recently, its expression has been noted in FGP Type II cells also, although a role in differentiation has not been tested [103]. HES1, a Notch pathway target and transcription factor, was observed to maintain the undifferentiated state of TB progenitors in CVP and to repress the transcription of Type II taste cells in developing immature taste cells [104]. A recent lingual epithelium RNA-sequencing study found *Hes1* in FGP [105], but its role in the anterior tongue has yet to be determined. In sum, there are varied regulatory pathways for TB progenitors in both FGP and CVP. The differences in roles of signaling pathways between FGP and CVP further underscore distinctions between anterior and posterior tongue regions. Much detail is lacking for in-depth knowledge of any of these pathways in either papilla type, and this impedes understanding of regulatory mechanisms for overall lingual functions. 

## 4. HH Signaling Regulation in Anterior and Posterior Tongue 

HH signaling is essential in the regulation of several tissues and organs in adults [106] including the tongue [1,2]. However, there is limited discussion for the varied effects in anterior and posterior tongue, and therefore, we emphasize this pathway. Briefly, HH signaling initiates through ligand binding to the canonical receptor Patched 1 (PTCH1), relieving inhibition of another membrane protein Smoothened (SMO). SMO mediates a signal transduction cascade that concludes with the modulation of GLI transcription factor activity and HH target genes including *Gli1* transcription [107]. In terms of the differences observed in the FGP and CVP from the structural to the molecular level, it is likely that their signaling regulation might be different. Therefore, we analyzed individual HH pathway components to compare the parallel expression in FGP and CVP cells. 

**Figure 3:**
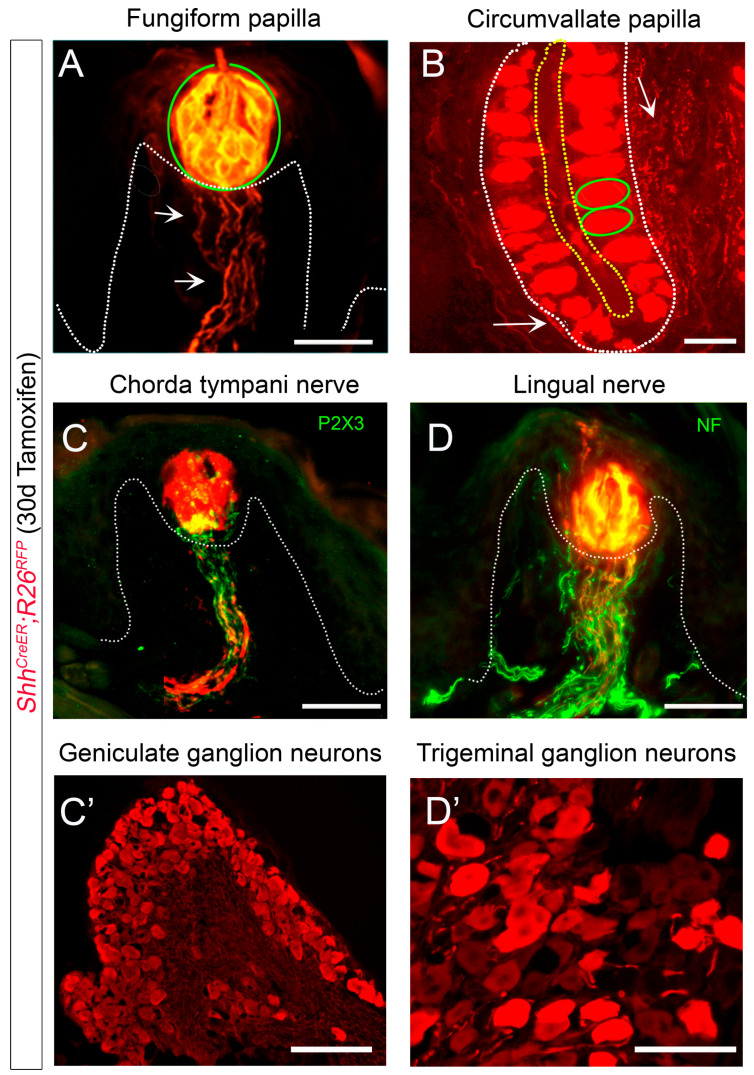
SHH expression in lingual papilla taste buds, nerves, and ganglia. (**A**–**D’**). RFP (red) expression after 30 days of tamoxifen administration in *Shh^CreER^;R26^RFP^* mice. (**A**,**B**). In both the anterior tongue fungiform (**A**) and posterior circumvallate (**B**) papillae, taste buds include *Shh*+ cells and their progeny (green oval outlines), while the connective tissue core includes *Shh*+ nerve fibers (white arrows). (**C**–**D’**). Chorda tympani nerve fibers ((**C**), P2X3+, green) and cell bodies in the geniculate ganglion (**C’**) are *Shh*+. Although all lingual nerve fibers ((**D**), NF+, green) do not express *Shh*, cell bodies in the trigeminal ganglion (**D’**) are *Shh*+. White dotted lines demarcate the basal lamina in subfigures (**A**–**D**). Yellow dotted line marks the apical taste buds regions in the papilla cleft in subfigure (**B**). Scale bar: 50 μm, applies to all images.

The SHH ligand is observed in Type IV basal taste cells, as well as in CT and GL nerves that enter TB in FGP and CVP, respectively [7,23,74,108,109]. SHH+ Type IV basal cells serve as an immediate precursor of all taste cell types [110]. Lineage tracing of *Shh*+ cells showed expression in all TB cells of FGP and CVP (Figure 3A,B) [23,108,109,110]. Further, *Shh*-positive expression co-labels with all CT fibers (P2X3+, Figure 3C) [111] and is present in neurons of the geniculate ganglion, the soma for the CT afferents (Figure 3C’) [23,108]. The *Shh*+ label does not completely overlap with LN fibers (NF+) (Figure 3D) [111] but is seen in LN innervation. Additionally, the trigeminal ganglion does have *Shh*+ cell bodies (Figure 3D’) [23,108]. Although GL nerves have *Shh*+ fibers (Figure 3B) [109], the petrosal ganglion has not been carefully investigated for the presence of SHH+ cells. 

**Figure 4:**
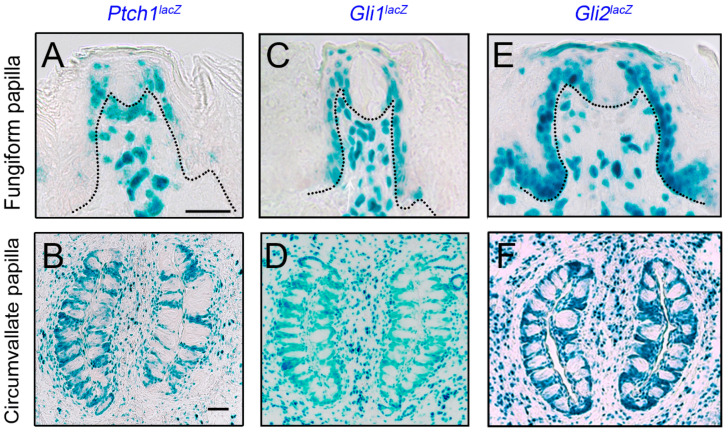
Hedgehog signaling components in anterior and posterior tongue papillae. (**A**–**F**). X-Gal staining (blue) for *Ptch1^lacZ^* (**A**,**B**), *Gli1^lacZ^* (**C**,**D**), and *Gli2^lacZ^* (**E**,**F**) mice in taste fungiform (**A**,**C**,**E**) and circumvallate (**B**,**D**,**F**) papillae. *Ptch1* and *Gli1* are expressed in papilla basal epithelial, perigemmal, and stromal cells, while *Gli2* is additionally present in anterior tongue non-taste, filiform basal epithelial and stromal cells. Black dotted lines in (**A**,**C**,**E**) demarcate the basal lamina. Scale bar (50 μm) in (**A**) applies to (**A**,**C**,**E**) and in (**B**) applies to (**B**,**D**,**F**).

The receptor *Ptch1* and HH-target gene *Gli1* are expressed in both anterior and posterior taste organs, in basal epithelial, perigemmal, and stromal cells (Figure 4A–D) [23,74,112,113,114]. Paracrine signaling has been suggested in the FGP taste organ that originates from TB SHH+ basal cells and signals to FGP basal, perigemmal, and stromal cells [74]. In addition to the above components, the main transcription activator of HH signaling, *Gli2* is noted in the entire lingual epithelium and stromal cells, including FGP and CVP (Figure 4E,F) [113]. Although the expression coincides with HH-responding *Gli1* cells in the CVP, in the anterior tongue, the *Gli1* target gene is absent from the non-taste filiform papillae epithelium. Overall, the expression of HH pathway components strongly suggests that the signaling is active in both FGP and CVP epithelium and stroma (Figure 4). 

Our studies with the epithelial-specific blockade of HH signaling showed elimination of TBs along with loss of SHH- and HH-responding *Gli1*+ epithelial cells in the anterior FGP [23,88]. However, even in the absence of epithelial SHH, lingual innervation and a few *Gli1*+ cells were retained in the FGP stroma. With the discovery of *Shh* in nerves, there is a possibility that paracrine signaling occurs in the connective tissue core, from *Shh*+ nerves to HH-responding stromal cells. In another study, deletion of SHH ligand from either lingual epithelium or gustatory neurons minimally affected TBs, while their combined loss abolished TBs [108]. Further, the authors showed that *Gli1* mRNA expression was intact in the lingual epithelium after epithelial SHH loss, while the expression was reduced, albeit not significantly, after neural SHH elimination and neural paracrine signaling within the TB was proposed. This is in contrast to our findings where epithelial HH signaling is lost even though nerves are retained [7,23,88,112]. A similar investigation into CVP has not been done. 

**Figure 5:**
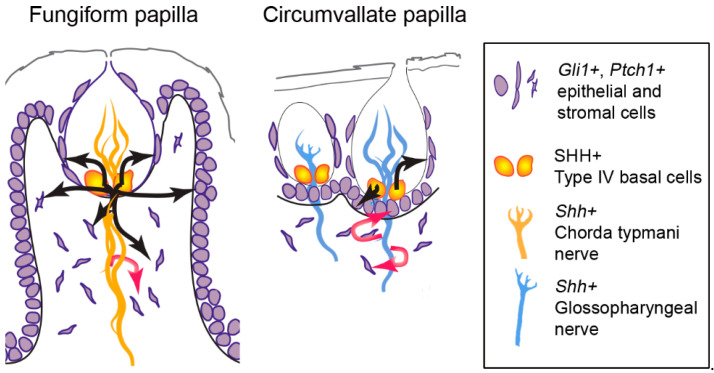
Paracrine SHH signaling in taste papillae epithelium and stroma. SHH ligand is present in Type IV basal cells and signals to *Gli1+* and *Ptch1+* HH-responding cells in the anterior tongue fungiform and posterior circumvallate papilla epithelium and stroma. Black arrows indicate epithelial paracrine signaling, and red arrows indicate neural paracrine signaling. In the fungiform papilla, epithelial SHH drives signaling in basal cells, perigemmal cells, and stromal cells (black arrows), while neural SHH signals to stromal cells (red arrow) (Note that neural paracrine signaling within the taste bud also has been suggested [108]). On the other hand, in the circumvallate papilla, epithelial SHH-responding targets are predominantly basal and perigemmal cells (black arrows), while neural SHH can target both basal and stromal cells (red arrows).

Importantly, after pharmacologic and genetic inhibition of HH signaling, the CVP TBs were reduced in number but not eliminated [10,23,88,115]. As a result, SHH expression within the TB was substantially reduced but not completely absent [10,88,115]. Intriguingly, unlike FGP with epithelial blockade of HH signaling, HH-responding *Gli1*+ cells in the CVP stroma were intact [23,88], suggesting a major contribution of *Shh*+ gustatory nerves in mediating paracrine signaling in CVP stroma. Further, when the GL nerve was bilaterally transected, there was rapid loss of *Shh* and *Ptch1* expression in the epithelium even though TBs were not degenerated [114,116]. In addition, after the GL nerve fibers regenerated, expression of epithelial *Shh* and *Ptch1* reappeared, reflecting direct control of epithelial HH signaling by the taste GL nerve in CVP [116]. In contrast, in the anterior tongue, when a few mm of CT/LN nerve were removed, smaller TBs with reduced TB-associated SHH were observed after 21 days of nerve cut [113]. In those atypical FGP, the remaining CT/LN fibers in the FGP connective tissue core were associated with *Gli1* and *Ptch1* cells [111], reiterating *Shh*+ nerve-mediated stromal HH signaling in FGP, but *Shh*+ nerve-independent FGP epithelial HH signaling. Overall, based on the source and papilla, the ligand SHH+ can have different roles in mediating FGP and CVP signaling. That is, within FGP epithelia SHH can initiate signaling to basal cells, perigemmal cells, and stromal cells (Figure 5 fungiform papilla, black arrows) while in CVP, epithelial SHH drives signaling predominantly in basal and perigemmal cells only (Figure 5 circumvallate papilla, black arrows). On the other hand, neural SHH can signal to local stromal cells in FGP (Figure 5 fungiform papilla, red arrow) and additionally in CVP epithelial cells (Figure 5 circumvallate papilla, red arrows). 

HH signaling has established roles in cell proliferation and differentiation of many organs [106]. Proliferation and differentiation require correct cell-specific signaling and a clear distinction from neighboring cells that should not be responding to these signals. As HH pathway inhibition resulted in a different extent of TB loss in FGP and CVP, it is likely that these functions may be differently affected in the anterior and posterior tongue. SMO antagonist treatment for 5–36 days was associated with reduction in cell proliferation at the FGP apical region [23,109], while CVP cell proliferation was maintained even after 36 days of treatment [10]. In contrast, another HH pathway inhibiting drug, vismodegib, after a 15-week treatment, led to reduction in CVP cell proliferation [115], suggesting that CVP might require longer continuous pathway inhibition treatment in mice to have proliferation disruption. Further, HH pathway inhibition with the drug sonidegib for 16–36 days resulted in virtual elimination of all taste cell types [23]. In CVP, only taste cell Type II was studied, and it was found to be significantly reduced after 15 weeks of treatment [115]. Overall, these studies indicate that HH signaling differentially regulates FGP and CVP proliferation and taste cell differentiation. That is, greater and faster effects of HH signaling were seen on FGP proliferation as compared to CVP. In addition, there was no increase in FGP taste cell death, whereas data suggest that TB cells lived their life cycle and were eliminated without getting replaced. Further, the HH pathway blockade inhibited TB differentiation, leading to the elimination of all TB cell types in FGP, but in CVP, the presence of a reduced number of Type II taste cells indicates maintenance of TB differentiation, although at low levels. Additional factors/pathways might be contributing to TB differentiation in CVP.

So far, most signaling regulation studies have been conducted in mice due to the availability of several reporter models, and differences are noted in the molecular signatures of FGP and CVP cells. Interestingly, in rats, a short-term pharmacological blockade of the HH pathway with sonidegib (16 days of treatment) led to complete loss of TBs in both FGP and CVP [10]. Thus, our findings strongly suggest that the differences or similarities in FGP and CVP regulation are further species-specific. Using the rat model, we recently found that pharmacological inhibition of the HH pathway by the cancer treatment drug sonidegib induced CT terminal field volume expansion in the brainstem, but GL fields remained intact [117]. Although there are limited numbers of studies of rat anterior versus posterior tongue in the context of HH signaling, there is clear knowledge that the HH pathway substantially regulates tongue homeostasis in FGP and CVP taste organs.

## 5. HH Antagonism and Regenerative Capacity of Anterior and Posterior Taste Organs

**Figure 6:**
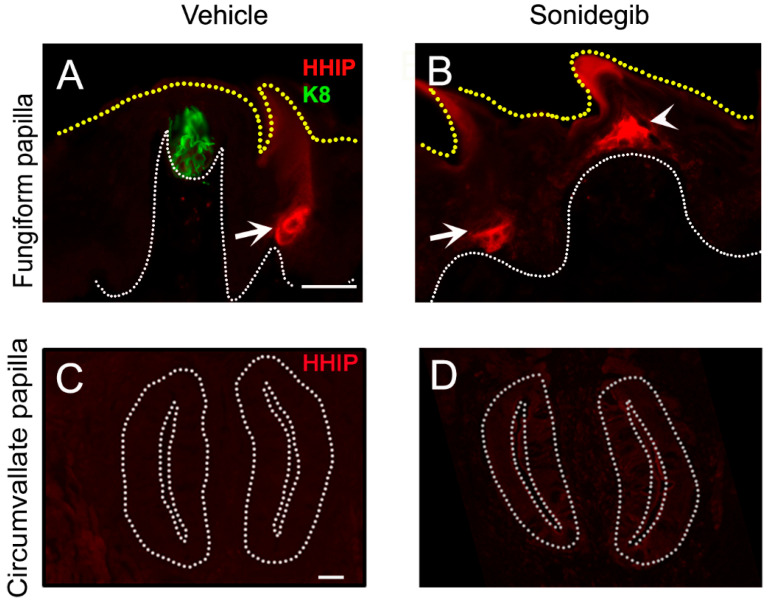
HH antagonist HHIP expression in fungiform and circumvallate papillae. (**A**–**D**) Antibody detection of endogenous HHIP (red) after vehicle (**A**,**C**) and sonidegib (**B**,**D**) treatment. (**A**,**B**) In the anterior tongue papillae, HHIP is expressed in the non-taste filiform papilla (white arrow) but not in the fungiform papilla or within the taste bud (green, K8) (**A**). After HH pathway inhibition with sonidegib, HHIP is ectopically expressed at the fungiform papilla apex (white arrowhead) (**B**). (**C**,**D**) In the circumvallate papilla there is no HHIP expression in the vehicle tongue (**C**). Unlike the fungiform papilla, ectopic HHIP is not observed after sonidegib exposure (**D**). White dotted lines demarcate basal lamina and yellow dotted lines mark the tongue surface in subfigures (**A**,**B**). White dotted lines in subfigures (**C**,**D**) outline the circumvallate papilla walls. Scale bar (50 μm) in (**A**) applies to (**A**,**B**) and (**C**) applies to (**C**,**D**).

Levels of HH signaling are maintained by pathway antagonists. The HH pathway target genes and antagonists HH-interacting protein (HHIP) and PTCH2 compete with PTCH1 for the ligand and lead to feedback inhibition of the signaling [118]. Notably, our recent work has revealed distinct HH-binding antagonist expression in the filiform papillae compared to FGP [113]. We observed Hhip (Figure 6A,B) and Ptch1 expression in the anterior epithelial face of the entire filiform papilla including those surrounding the FGP in mouse models [113]. The presence of HHIP cannot be evaluated in rats due to the lack of validated antibodies or reporter models. Notably, as the CVP does not have adjacent typical filiform papillae (although there are large epithelial ‘plates’ around the CVP), Hhip, both the gene and protein, was not observed in the posterior tongue (Figure 6C) [113]. The findings also indicate that the transmembrane receptor PTCH1 potentially plays a dual role in the distinct locations of the tongue, as a SHH ligand receptor in FGP and CVP but as an antagonist in filiform epithelium. Together, the data suggest that the HH pathway regulates the anterior tongue in a different manner than the posterior tongue. Levels of HH signaling are controlled by HH antagonism regulation in the anterior tongue but not in the posterior tongue. The posterior tongue might have cross-talk with additional pathways to achieve signaling controls.

Strikingly, when the HH pathway was inhibited, either pharmacologically with the SMO-inhibiting drug, sonidegib, or by using *Smo* and *Gli2* genetic deletion mouse models, HHIP (Figure 6B) and *Ptch1* were ectopically expressed in the FGP apex, which becomes conical [113], but not in the CVP (Figure 6D) [119]. The differential expression of antagonist might be associated with significant TB loss in FGP but a comparatively lesser TB loss in CVP even after long-term HH pathway inhibition (48 days) in the mouse model [10]. Importantly, HH pathway inhibition did not alter the filiform papillae or HHIP expression (Figure 6A,B, white arrows), suggesting an HH-independent expression of HH antagonists in filiform papillae [113]. In contrast, mis-activation of SHH in the entire lingual epithelium was associated with TB formation in the non-gustatory filiform papilla epithelium [108,109]. The posterior tongue was not studied after over-activation of the HH pathway. Thus, even though the expressions of HH pathway components are similar in both FGP and CVP, disruption of HH signaling causes a different extent of tissue disruption in the anterior and posterior tongue. The FGP lost its TBs and rectangular structural appearance with simultaneous induction of HH antagonists, while the CVP structure was maintained with modest loss of TBs and without any subsequent HH antagonist expression (Figure 6B,D).

As noted, we revealed differential loss of TBs in FGP and CVP after pharmacologic or genetic blockade of the HH pathway [10,23,88,112]. To study the regenerative potential of the anterior vs. posterior taste organ, after treating mice with the HH pathway inhibition drug, sonidegib, for short-term (16 days) and long-term (48 days) periods, we discontinued the drug treatment and allowed the animals to recover for several days (7, 14, and 21) or months (3, 5, 7, and 9). Short-term pathway blockade caused the virtual elimination of typical taste organs in FGP with a simultaneous increase in atypical taste organs with or without the TB, while CVP had a modest loss of typical taste pores [23]. Moreover, after 48 days of sonidegib treatment, the majority of FGP were without any TB, while one-fifth of typical taste pores still remained in the CVP [10]. When allowed to recover, CVP completely regenerated all the TB profiles with intact typical taste pores after both short-term and long-term HH pathway inhibition. On the other hand, only atypical FGP with TB remnants recovered to typical taste organs, while atypical FGP without any TB could not recover even after 9 months of recovery [10,23]. Thus, a partial recovery of FGP TBs was noted after short-term HH pathway inhibition, while after long-term inhibition, there was no recovery of FGP TBs. Intriguingly, atypical FGP that were unable to recover had ectopic HHIP expression and thus could not resume HH signaling [113]. This is critical as the presence of an antagonist in FGP prevented taste organ regeneration. On the other hand, sonidegib did not induce ectopic HHIP expression in CVP (Figure 6D), which might have allowed for complete recovery of CVP TBs. Our data suggest that HH signaling and antagonism play a highly significant role in anterior FGP taste organ homeostasis and renewal as compared to posterior CVP in mouse. 

## 6. Conclusions

It is crucial in the field to understand cellular and molecular regional differences in tongue taste organs to delineate genetic and environmental roles in taste and somatosensory sensations that are associated with eating and other major oral functions. We emphasize that having information about both anterior and posterior tongue taste organs and signaling regulation is essential to interpret structural, neurophysiological, and behavioral data. In the mouse model, our studies with HH pathway inhibition revealed elimination of TBs in FGP but not in CVP. Further, the taste cell types that were retained in the remaining TB cells in the CVP have not yet been determined. This knowledge would contribute to design of better management of taste disturbances. Thus, we reiterate that analysis of one taste organ only will provide an incomplete picture of the differential participation of tongue regions in all lingual functions. Furthermore, to fully understand genes that participate in taste signaling and other sensory functions, it is essential to study papillae, TBs, and ganglia associated with the anterior and posterior tongue regions. 

## Data Availability

Data sharing not applicable.

## Abbreviations

CT	Chorda tympani
CVP	Circumvallate papillae
FGP	Fungiform papillae
FOP	Foliate papillae
GL	Glossopharyngeal
GPCR	G-protein-coupled receptor
HH	Hedgehog
HHIP	HH-interacting protein
LGR	Leucine-rich repeat-containing G-protein-coupled receptor
LN	Lingual
PTCH	Patched
SHH	Sonic HH
SMO	Smoothened
TB	Taste bud
TRP	Transient receptor potential

## References

[1] Mistretta C.M., Kumari A. (2017). Tongue and Taste Organ Biology and Function: Homeostasis Maintained by Hedgehog Signaling. Annu. Rev. Physiol..

[2] Mistretta C.M., Kumari A. (2019). Hedgehog Signaling Regulates Taste Organs and Oral Sensation: Distinctive Roles in the Epithelium, Stroma, and Innervation. Int. J. Mol. Sci..

[3] Schier L.A., Spector A.C. (2019). The Functional and Neurobiological Properties of Bad Taste. Physiol. Rev..

[4] Rothova M., Thompson H., Lickert H., Tucker A.S. (2012). Lineage Tracing of the Endoderm during Oral Development. Dev. Dyn..

[5] Doyle M.E., Premathilake H.U., Yao Q., Mazucanti C.H., Egan J.M. (2023). Physiology of the tongue with emphasis on taste transduction. Physiol. Rev..

[6] Kessler A.T., Bhatt A.A. (2018). Review of the Major and Minor Salivary Glands, Part 1: Anatomy, Infectious, and Inflammatory Processes. J. Clin. Imaging Sci..

[7] Donnelly C.R., Kumari A., Li L., Vesela I., Bradley R.M., Mistretta C.M., Pierchala B.A. (2021). Probing the Multimodal Fungiform Papilla: Complex Peripheral Nerve Endings of Chorda Tympani Taste and Mechanosensitive Fibers before and after Hedgehog Pathway Inhibition. Cell Tissue Res..

[8] Mistretta C.M., Bradley R.M. (2021). The Fungiform Papilla Is a Complex, Multimodal, Oral Sensory Organ. Curr. Opin. Physiol..

[9] Simon E., Mertens P. (2009). Functional Anatomy of the Glossopharyngeal, Vagus, Accessory and Hypoglossal Cranial Nerves. Neurochirurgie.

[10] Kumari A., Yokota Y., Li L., Bradley R.M., Mistretta C.M. (2018). Species Generalization and Differences in Hedgehog Pathway Regulation of Fungiform and Circumvallate Papilla Taste Function and Somatosensation Demonstrated with Sonidegib. Sci. Rep..

[11] Donnelly C.R., Shah A.A., Mistretta C.M., Bradley R.M., Pierchala B.A. (2018). Biphasic Functions for the GDNF-Ret Signaling Pathway in Chemosensory Neuron Development and Diversification. Proc. Natl. Acad. Sci. USA.

[12] Dvoryanchikov G., Hernandez D., Roebber J.K., Hill D.L., Roper S.D., Chaudhari N. (2017). Transcriptomes and Neurotransmitter Profiles of Classes of Gustatory and Somatosensory Neurons in the Geniculate Ganglion. Nat. Commun..

[13] Moayedi Y., Xu S., Obayashi S.K., Hoffman B.U., Gerling G.J., Lumpkin E.A. (2022). In Vivo Calcium Imaging Identifies Functionally and Molecularly Distinct Subsets of Tongue-Innervating Mechanosensory Neurons. bioRxiv.

[14] Yokota Y., Bradley R.M. (2016). Receptive Field Size, Chemical and Thermal Responses, and Fiber Conduction Velocity of Rat Chorda Tympani Geniculate Ganglion Neuron. J. Neurophysiol..

[15] Yokota Y., Bradley R.M. (2017). Geniculate Ganglion Neurons are Multimodal and Variable in Receptive Field Characteristics. Neuroscience.

[16] Aps J.K., Martens L.C. (2005). The Physiology of Saliva and Transfer of Drugs into Saliva. Forensic. Sci. Int..

[17] Rao A., Tadi P. (2022). Anatomy, Head and Neck, Chorda Tympani. StatPearls.

[18] D’Silva N.J., Perez-Pacheco C., Schmitd L.B. (2022). The 3D’s of Neural Phenotypes in Oral Cancer: Distance, Diameter, and Density. Adv. Biol..

[19] Thomas K., Minutello K., Das J.M. (2022). Neuroanatomy, Cranial Nerve 9 (Glossopharyngeal). StatPearls.

[20] Dvoryanchikov G., Tomchik S.M., Chaudhari N. (2007). Biogenic Amine Synthesis and Uptake in Rodent Taste Buds. J. Comp. Neurol..

[21] Tang T., Pierchala B.A. (2022). Oral Sensory Neurons of the Geniculate Ganglion That Express Tyrosine Hydroxylase Comprise a Subpopulation That Contacts Type II and Type III Taste Bud Cells. eNeuro.

[22] Kim S., Naqvi I. (2022). Neuroanatomy, Cranial Nerve 12 (Hypoglossal). StatPearls.

[23] Kumari A., Ermilov A.N., Grachtchouk M., Dlugosz A.A., Allen B.L., Bradley R.M., Mistretta C.M. (2017). Recovery of Taste Organs and Sensory Function after Severe Loss from Hedgehog/Smoothened Inhibition with Cancer Drug Sonidegib. Proc. Natl. Acad. Sci. USA.

[24] Danilova V., Hellekant G. (2003). Comparison of the Responses of the Chorda Tympani and Glossopharyngeal Nerves to Taste Stimuli in C57BL/6J Mice. BMC. Neurosci..

[25] Barlow L.A., Klein O.D. (2015). Developing and Regenerating a Sense of Taste. Curr. Top. Dev. Biol..

[26] Finger T.E., Barlow L.A. (2021). Cellular Diversity and Regeneration in Taste Buds. Curr. Opin. Physiol..

[27] Gaillard D., Barlow L.A. (2021). A Mechanistic Overview of Taste Bud Maintenance and Impairment in Cancer Therapies. Chem. Senses.

[28] von Molitor E., Riedel K., Krohn M., Hafner M., Rudolf R., Cesetti T. (2021). Sweet Taste Is Complex: Signaling Cascades and Circuits Involved in Sweet Sensation. Front. Hum. Neurosci..

[29] Taruno A., Nomura K., Kusakizako T., Ma Z., Nureki O., Foskett J.K. (2021). Taste Transduction and Channel Synapses in Taste Buds. Pflugers. Arch..

[30] Ogata T., Ohtubo Y. (2020). Quantitative Analysis of Taste Bud Cell Numbers in the Circumvallate and Foliate Taste Buds of Mice. Chem. Senses.

[31] Boughter J.D., Pumplin D.W., Yu C., Christy R.C., Smith D.V. (1997). Differential Expression of α-Gustducin in Taste Bud Populations of the Rat and Hamster. J. Neurosci..

[32] Ohman L.C., Krimm R.F. (2021). Whole-Mount Staining, Visualization, and Analysis of Fungiform, Circumvallate, and Palate Taste Buds. J. Vis. Exp. JoVE.

[33] Roper S.D., Chaudhari N. (2017). Taste Buds: Cells, Signals and Synapses. Nat. Rev. Neurosci..

[34] Caicedo A., Kim K.N., Roper S.D. (2002). Individual Mouse Taste Cells Respond to Multiple Chemical Stimuli. J. Physiol..

[35] Kimura K., Beidler L.M. (1961). Microelectrode Study of Taste Receptors of Rat and Hamster. J. Cell. Comp. Physiol..

[36] Sato T., Beidler L.M. (1997). Broad Tuning of Rat Taste Cells for Four Basic Taste Stimuli. Chem. Senses..

[37] Tonosaki K., Funakoshi M. (1984). The Mouse Taste Cell Response to Five Sugar Stimuli. Comp. Biochem. Physiol. A Comp. Physiol..

[38] Rodriguez Y.A., Roebber J.K., Dvoryanchikov G., Makhoul V., Roper S.D., Chaudhari N. (2021). “Tripartite Synapses” in Taste Buds: A Role for Type I Glial-like Taste Cells. J. Neurosci..

[39] Dutta Banik D., Martin L.E., Freichel M., Torregrossa A.M., Medler K.F. (2018). TRPM4 and TRPM5 are Both Required for Normal Signaling in Taste Receptor Cells. Proc. Natl. Acad. Sci. USA.

[40] Zhang Y., Hoon M.A., Chandrashekar J., Mueller K.L., Cook B., Wu D., Zuker C.S., Ryba N.J.P. (2003). Coding of Sweet, Bitter, and Umami Tastes: Different Receptor Cells Sharing Similar Signaling Pathways. Cell.

[41] Wooding S.P., Ramirez V.A., Behrens M. (2021). Bitter Taste Receptors: Genes, Evolution and Health. Evol. Med. Public Health.

[42] Yamada Y., Takai S., Watanabe Y., Osaki A., Kawabata Y., Oike A., Hirayama A., Iwata S., Sanematsu K., Tabata S. (2021). Gene Expression Profiling of a-Gustducin-Expressing Taste Cells in Mouse Fungiform and Circumvallate Papillae. Biochem. Biophys. Res. Commun..

[43] Yoshida R., Miyauchi A., Yasuo T., Jyotaki M., Murata Y., Yasumatsu K., Shigemura N., Yanagawa Y., Obata K., Ueno H. (2009). Discrimination of Taste Qualities among Mouse Fungiform Taste Bud Cells. J. Physiol..

[44] Chandrashekar J., Hoon M.A., Ryba N.J., Zuker C.S. (2006). The Receptors and Cells for Mammalian Taste. Nature.

[45] Lewandowski B.C., Sukumaran S.K., Margolskee R.F., Bachmanov A.A. (2016). Amiloride-Insensitive Salt Taste Is Mediated by Two Populations of Type III Taste Cells with Distinct Transduction Mechanisms. J. Neurosci..

[46] Tomchik S.M., Berg S., Kim J.W., Chaudhari N., Roper S.D. (2007). Breadth of Tuning and Taste Coding in Mammalian Taste Buds. J. Neurosci..

[47] Dutta Banik D., Benfey E.D., Martin L.E., Kay K.E., Loney G.C., Nelson A.R., Ahart Z.C., Kemp B.T., Kemp B.R., Torregrossa A.-M. (2020). A Subset of Broadly Responsive Type III Taste Cells Contribute to the Detection of Bitter, Sweet and Umami Stimuli. PLOS Genet..

[48] Vandenbeuch A., Clapp T.R., Kinnamon S.C. (2008). Amiloride-Sensitive Channels in Type I Fungiform Taste Cells in Mouse. BMC Neurosci..

[49] Wilson C.E., Finger T.E., Kinnamon S.C. (2017). Type III Cells in Anterior Taste Fields Are More Immunohistochemically Diverse Than Those of Posterior Taste Fields in Mice. Chem. Senses.

[50] Doolin R.E., Gilbertson T.A. (1996). Distribution and Characterization of Functional Amiloride-Sensitive Sodium Channels in Rat Tongue. J. Gen. Physiol..

[51] Lin W., Finger T.E., Rossier B.C., Kinnamon S.C. (1999). Epithelial Na+ Channel Subunits in Rat Taste Cells: Localization and Regulation by Aldosterone. J. Comp. Neurol..

[52] Kretz O., Barbry P., Bock R., Lindemann B. (1999). Differential Expression of RNA and Protein of the Three Pore-forming Subunits of the Amiloride-sensitive Epithelial Sodium Channel in Taste Buds of the Rat. J. Histochem. Cytochem..

[53] Hoon M.A., Adler E., Lindemeier J., Battey J.F., Ryba N.J.P., Zuker C.S. (1999). Putative Mammalian Taste Receptors: A Class of Taste-Specific GPCRs with Distinct Topographic Selectivity. Cell.

[54] Nelson G., Hoon M.A., Chandrashekar J., Zhang Y., Ryba N.J.P., Zuker C.S. (2001). Mammalian Sweet Taste Receptors. Cell.

[55] Voigt A., Hübner S., Lossow K., Hermans-Borgmeyer I., Boehm U., Meyerhof W. (2012). Genetic Labeling of Tas1r1 and Tas2r131 Taste Receptor Cells in Mice. Chem. Senses.

[56] Stone L.M., Barrows J., Finger T.E., Kinnamon S.C. (2007). Expression of T1Rs and Gustducin in Palatal Taste Buds of Mice. Chem. Senses.

[57] Montmayeur J.P., Liberles S.D., Matsunami H., Buck L.B. (2001). A Candidate Taste Receptor Gene Near a Sweet Taste Locus. Nat. Neurosci..

[58] Adler E., Hoon M.A., Mueller K.L., Chandrashekar J., Ryba N.J.P., Zuker C.S. (2000). A Novel Family of Mammalian Taste Receptors. Cell.

[59] Lossow K., Hübner S., Roudnitzky N., Slack J.P., Pollastro F., Behrens M., Meyerhof W. (2016). Comprehensive Analysis of Mouse Bitter Taste Receptors Reveals Different Molecular Receptive Ranges for Orthologous Receptors in Mice and Humans. J. Biol. Chem..

[60] Shindo Y., Miura H., Carninci P., Kawai J., Hayashizaki Y., Ninomiya Y., Hino A., Kanda T., Kusakabe Y. (2008). Gα14 is a Candidate Mediator of Sweet/Umami Signal Transduction in the Posterior Region of the Mouse Tongue. Biochem. Biophys. Res. Commun..

[61] Tizzano M., Dvoryanchikov G., Barrows J.K., Kim S., Chaudhari N., Finger T.E. (2008). Expression of Galpha14 In Sweet-Transducing Taste Cells of the Posterior Tongue. BMC Neurosci..

[62] Clapp T.R., Yang R., Stoick C.L., Kinnamon S.C., Kinnamon J.C. (2004). Morphologic Characterization of Rat Taste Receptor Cells that Express Components of the Phospholipase C Signaling Pathway. J. Comp. Neurol..

[63] DeFazio R.A., Dvoryanchikov G., Maruyama Y., Kim J.W., Pereira E., Roper S.D., Chaudhari N. (2006). Separate Populations of Receptor Cells and Presynaptic Cells in Mouse Taste Buds. J. Neurosci..

[64] Huang A.L., Chen X., Hoon M.A., Chandrashekar J., Guo W., Tränkner D., Ryba N.J., Zuker C.S. (2006). The Cells and Logic for Mammalian Sour Taste Detection. Nature.

[65] Horio N., Yoshida R., Yasumatsu K., Yanagawa Y., Ishimaru Y., Matsunami H., Ninomiya Y. (2011). Sour Taste Responses in Mice Lacking PKD Channels. PLoS ONE.

[66] Lossow K., Hermans-Borgmeyer I., Behrens M., Meyerhof W. (2017). Genetic Labeling of Car4-expressing Cells Reveals Subpopulations of Type III Taste Cells. Chem. Senses.

[67] Koyanagi-Matsumura E., Miura H., Saito M., Harada S. (2021). Type II/III Cell Composition and NCAM Expression in Taste Buds. Cell Tissue Res..

[68] Farbman A.I. (1965). Fine Structure of the Taste Bud. J. Ultrastruct. Res..

[69] Beidler L.M., Smallman R.L. (1965). Renewal of Cells within Taste Bud. J. Cell Biol..

[70] Delay R.J., Kinnamon J.C., Roper S.D. (1986). Ultrastructure of Mouse Vallate Taste Buds: II. Cell Types and Cell Lineage. J. Comp. Neurol..

[71] Hamamichi R., Asano-Miyoshi M., Emori Y. (2006). Taste Bud contains both Short-Lived and Long-Lived Cell Populations. Neuroscience.

[72] Farbman A.I. (1980). Renewal of Taste Bud Cells in Rat Circumvallate Papillae. Cell Tissue Kinet..

[73] Perea-Martinez I., Nagai T., Chaudhari N. (2013). Functional Cell Types in Taste Buds Have Distinct Longevities. PLoS ONE.

[74] Liu H.X., Ermilov A., Grachtchouk M., Li L., Gumucio D.L., Dlugosz A.A., Mistretta C.M. (2013). Multiple Shh Signaling Centers Participate in Fungiform Papilla and Taste Bud Formation and Maintenance. Dev. Biol..

[75] Okubo T., Clark C., Hogan B.L. (2009). Cell Lineage Mapping of Taste Bud Cells and Keratinocytes in The Mouse Tongue and Soft Palate. Stem. Cells.

[76] Miura H., Kusakabe Y., Harada S. (2006). Cell Lineage and Differentiation in Taste Buds. Arch. Histol. Cytol..

[77] Nguyen H.M., Barlow L.A. (2010). Differential Expression of a BMP4 Reporter Allele in Anterior Fungiform Versus Posterior Circumvallate Taste Buds of Mice. BMC Neurosci..

[78] Ren W., Lewandowski B.C., Watson J., Aihara E., Iwatsuki K., Bachmanov A.A., Margolskee R.F., Jiang P. (2014). Single Lgr5- Or Lgr6-Expressing Taste Stem/Progenitor Cells Generate Taste Bud Cells Ex Vivo. Proc. Natl. Acad. Sci. USA.

[79] Takeda N., Jain R., Li D., Li L., Lu M.M., Epstein J.A. (2013). Lgr5 Identifies Progenitor Cells Capable of Taste Bud Regeneration after Injury. PLoS ONE.

[80] Yee K.K., Li Y., Redding K.M., Iwatsuki K., Margolskee R.F., Jiang P. (2013). Lgr5-EGFP Marks Taste Bud Stem/Progenitor Cells in Posterior Tongue. Stem. Cells.

[81] Hsu S.Y., Kudo M., Chen T., Nakabayashi K., Bhalla A., van der Spek P.J., van Duin M., Hsueh A.J.W. (2000). The Three Subfamilies of Leucine-Rich Repeat-Containing G Protein-Coupled Receptors (LGR): Identification of LGR6 and LGR7 and the Signaling Mechanism for LGR7. Mol. Endocrinol..

[82] Kumar K.K., Burgess A.W., Gulbis J.M. (2014). Structure and Function of LGR5: An Enigmatic G-Protein Coupled Receptor Marking Stem Cells. Protein. Sci..

[83] Ohmoto M., Lei W., Yamashita J., Hirota J., Jiang P., Matsumoto I. (2020). SOX2 Regulates Homeostasis of Taste Bud Cells and Lingual Epithelial Cells in Posterior Tongue. PLoS ONE.

[84] Castillo-Azofeifa D., Seidel K., Gross L., Golden E.J., Jacquez B., Klein O.D., Barlow L.A. (2018). SOX2 Regulation by Hedgehog Signaling Controls Adult Lingual Epithelium Homeostasis. Development.

[85] Ohmoto M., Ren W., Nishiguchi Y., Hirota J., Jiang P., Matsumoto I. (2017). Genetic Lineage Tracing in Taste Tissues Using Sox2-Creert2 Strain. Chem. Senses.

[86] Ohmoto M., Nakamura S., Wang H., Jiang P., Hirota J., Matsumoto I. (2022). Maintenance and Turnover of Sox2+ Adult Stem Cells in the Gustatory Epithelium. PLoS ONE.

[87] Barlow L.A. (2022). The Sense of Taste: Development, Regeneration, and Dysfunction. WIREs. Mech. Dis..

[88] Ermilov A.N., Kumari A., Li L., Joiner A.M., Grachtchouk M.A., Allen B.L., Dlugosz A.A., Mistretta C.M. (2016). Maintenance of Taste Organs is Strictly Dependent on Epithelial Hedgehog/GLI Signaling. PLoS. Genet..

[89] Qin Y., Sukumaran S.K., Margolskee R.F. (2021). Nkx2-2 Expressing Taste Cells in Endoderm-Derived Taste Papillae are Committed to The Type III Lineage. Dev. Biol..

[90] Seta Y., Toyono T., Takeda S., Toyoshima K. (1999). Expression of Mash1 in Basal Cells of Rat Circumvallate Taste Buds is Dependent upon Gustatory Innervation. FEBS Lett..

[91] Miura H., Kusakabe Y., Kato H., Miura-Ohnuma J., Tagami M., Ninomiya Y., Hino A. (2003). Co-Expression Pattern of Shh with Prox1 and that of Nkx2.2 with Mash1 in Mouse Taste Bud. Gene. Expr. Patterns.

[92] Hsu C.-C., Seta Y., Matsuyama K., Kataoka S., Nakatomi M., Toyono T., Gunjigake K.K., Kuroishi K.N., Kawamoto T. (2021). Mash1-Expressing Cells may be Relevant to Type III Cells and a Subset of Plcβ2-Positive Cell Differentiation in Adult Mouse Taste Buds. Cell Tissue Res..

[93] Seta Y., Oda M., Kataoka S., Toyono T., Toyoshima K. (2011). Mash1 is Required for the Differentiation of AADC-Positive Type III Cells in Mouse Taste Bud. Dev. Dyn..

[94] Seta Y., Stoick-Cooper C.L., Toyono T., Kataoka S., Toyoshima K., Barlow L.A. (2006). The bHLH Transcription Factors, Hes6 and Mash1, are Expressed in Distinct Subsets of Cells within Adult Mouse Taste Buds. Arch. Histol. Cytol..

[95] Takagi H., Seta Y., Kataoka S., Nakatomi M., Toyono T., Kawamoto T. (2018). Mash1-Expressing Cells could Differentiate to Type III Cells in Adult Mouse Taste Buds. Anat. Sci. Int..

[96] Qin Y., Sukumaran S.K., Jyotaki M., Redding K., Jiang P., Margolskee R.F. (2018). Gli3 is a Negative Regulator of Tas1r3-Expressing Taste Cells. PLoS Genet..

[97] Gaillard D., Barlow L.A. (2011). Taste Bud Cells of Adult Mice are Responsive to Wnt/Beta-Catenin Signaling: Implications for the Renewal of Mature Taste Cells. Genesis.

[98] Gaillard D., Xu M., Liu F., Millar S.E., Barlow L.A. (2015). β-Catenin Signaling Biases Multipotent Lingual Epithelial Progenitors to Differentiate and Acquire Specific Taste Cell Fates. PLoS Genet..

[99] Gaillard D., Bowles S.G., Salcedo E., Xu M., Millar S.E., Barlow L.A. (2017). β-catenin is Required for Taste Bud Cell Renewal and Behavioral Taste Perception in Adult Mice. PLoS Genet..

[100] Schneider F.T., Schanzer A., Czupalla C.J., Thom S., Engels K., Schmidt M.H.H., Plate K.H., Liebner S. (2010). Sonic Hedgehog Acts as a Negative Regulator of Beta-Catenin Signaling in the Adult Tongue Epithelium. Am. J. Pathol..

[101] Matsumoto I., Ohmoto M., Narukawa M., Yoshihara Y., Abe K. (2011). Skn-1a (Pou2f3) Specifies Taste Receptor Cell Lineage. Nat. Neurosci..

[102] Yamashita J., Ohmoto M., Yamaguchi T., Matsumoto I., Hirota J. (2017). Skn-1a/Pou2f3 Functions as a Master Regulator to Generate Trpm5-Expressing Chemosensory Cells in Mice. PLoS ONE.

[103] Ohmoto M., Kitamoto S., Hirota J. (2021). Expression of Eya1 in Mouse Taste Buds. Cell. and. Tissue. Research..

[104] Ota M.S., Kaneko Y., Kondo K., Ogishima S., Tanaka H., Eto K., Kondo T. (2009). Combined In Silico and In Vivo Analyses Reveal Role of Hes1 in Taste Cell Differentiation. PLoS Genet..

[105] Consortium T.M. (2018). Single-Cell Transcriptomics of 20 Mouse Organs Creates A Tabula Muris. Nature.

[106] Petrova R., Joyner A.L. (2014). Roles for Hedgehog Signaling in Adult Organ Homeostasis and Repair. Development.

[107] Briscoe J., Therond P.P. (2013). The Mechanisms of Hedgehog Signalling and Its Roles in Development and Disease. Nat. Rev. Mol. Cell Biol..

[108] Castillo-Azofeifa D., Losacco J.T., Salcedo E., Golden E.J., Finger T.E., Barlow L.A. (2017). Sonic Hedgehog from Both Nerves and Epithelium is a Key Trophic Factor for Taste Bud Maintenance. Development.

[109] Lu W.J., Mann R.K., Nguyen A., Bi T., Silverstein M., Tang J.Y., Chen X., Beachy P.A. (2018). Neuronal Delivery of Hedgehog Directs Spatial Patterning of Taste Organ Regeneration. Proc. Natl. Acad. Sci. USA.

[110] Miura H., Scott J.K., Harada S., Barlow L.A. (2014). Sonic Hedgehog-Expressing Basal Cells are General Post-Mitotic Precursors of Functional Taste Receptor Cells. Dev. Dyn..

[111] Mistretta C.M., Kumari A., Li L., Allen B.L., Bradley R.M. Nerves and Sonic Hedgehog Signaling Interactions in Fungiform Papilla Taste Organ Homeostasis. Proceedings of the Association for Chemoreception Sciences.

[112] Kumari A., Ermilov A.N., Allen B.L., Bradley R.M., Dlugosz A.A., Mistretta C.M. (2015). Hedgehog Pathway Blockade with the Cancer Drug LDE225 Disrupts Taste Organs and Taste Sensation. J. Neurophysiol..

[113] Kumari A., Li L., Ermilov A.N., Franks N.E., Dlugosz A.A., Allen B.L., Mistretta C.M. (2022). Hedgehog (HH) Pathway Endogenous Antagonist HHIP: Unique Lingual Expression in Filiform Papillae During Homeostasis and Ectopic in Fungiform Papillae during HH Signaling Inhibition. Dev. Dyn..

[114] Miura H., Kusakabe Y., Sugiyama C., Kawamatsu M., Ninomiya Y., Motoyama J., Hino A. (2001). Shh and Ptc are Associated with Taste Bud Maintenance in the Adult Mouse. Mech. Dev..

[115] Yang H., Cong W.N., Yoon J.S., Egan J.M. (2015). Vismodegib, an Antagonist of Hedgehog Signaling, Directly Alters Taste Molecular Signaling in Taste Buds. Cancer. Med..

[116] Miura H., Kato H., Kusakabe Y., Tagami M., Miura-Ohnuma J., Ninomiya Y., Hino A. (2004). A Strong Nerve Dependence of Sonic Hedgehog Expression in Basal Cells in Mouse Taste Bud and an Autonomous Transcriptional Control of Genes in Differentiated Taste Cells. Chem. Senses.

[117] Sun C., Kumari A., Mistretta C.M., Hill D. Reorganization of Gustatory Inputs Into the Nst Induced in Adult Rats by the Hedgehog Pathway Inhibitor, Sonidegib. Proceedings of the Association for Chemoreception Sciences.

[118] Chuang P.T., McMahon A.P. (1999). Vertebrate Hedgehog Signalling Modulated by Induction of a Hedgehog-Binding Protein. Nature.

[119] Kumari A., Mistretta C.M., Allen B.L. Taste Homeostasis And Regeneration: Hedgehog Signaling And Antagonism. Proceedings of the Association for Chemoreception Sciences.

